# Meta-Analysis of Clinical Efficacy and Safety of Ligustrazine in the Treatment of Idiopathic Pulmonary Fibrosis

**DOI:** 10.1155/2020/2416132

**Published:** 2020-11-06

**Authors:** Xiaozheng Wu, Wen Li, Zhenliang Luo, Yunzhi Chen

**Affiliations:** Department of Preclinical Medicine, Guizhou University of Traditional Chinese Medicine, Guiyang 510025, China

## Abstract

**Objective:**

To systematically review the efficacy and safety of Ligustrazine in the treatment of idiopathic pulmonary fibrosis (IPF).

**Methods:**

The electronic literature databases (PubMed, EMbase, CNKI, WanFang database, and VIP) were retrieved through a computer to find out the randomized controlled trials (RCT) of Ligustrazine in the treatment of IPF according to the inclusion/exclusion criteria screening test. Cochrane's bias risk table was also used to evaluate the quality of the study and to extract effective data. RevMan 5.3 was used for statistical analysis.

**Results:**

A total of 7 RCTs (a total of 366 patients, including 196 in experimental and 170 in control group). Compared with the control group, Ligustrazine could improve the clinical symptoms ([OR] = 2.20, 95% CI [1.40, 3.46], *P*=0.0006), lung function (VC % [MD] = 3.92, 95% CI [0.68, 7.17], *P*=0.02), (TLC% [MD] = 4.94, 95% CI [0.37, 9.52], *P*=0.03), the pulmonary diffusion function (DLCO % [MD] = 9.12, 95% CI [5.70, 12.55], *P* < 0.00001), and arterial blood gas analysis (PaO_2_ [MD] = 7.11, 95% CI [1.96, 12.25], *P*=0.007) (PaCO_2_ [MD] = −2.42, 95% CI [−4.36, −0.49], *P*=0.01) of IPF patients, respectively. However, FEV_1_/FVC % ([MD] = 9.37, 95% CI [−1.23, 19.97], *P*=0.08) and adverse reactions ([MD] = 0.35, 95% CI [0.02, 5.36], *P*=0.45) were not significantly improved.

**Conclusion:**

Ligustrazine has certain clinical efficacy in the treatment of IPF, but the safety of applying it and the adverse reactions need to be further analyzed and determined. It can be considered as a new alternative and complementary medicine to be promoted and recommended for use in medical units in various countries in the world and it solved the difficult problem of conventional drug treatment of IPF; therefore, more research strength can be put in the treatment of the pathological mechanism of IPF for further exploration. The study was registered under registration number CRD42020193626.

## 1. Introduction

Idiopathic pulmonary fibrosis (IPF) is a different type of chronic progressive pulmonary interstitial inflammation and fibrosis with unknown etiology [1]. In the past 20 years, its incidence has increased, with a median survival period of 3–5 years and a 5-year survival rate of 20–40% [1]. At present, the mainstream view of its pathogenesis is that the abnormal damage-repair process involving pulmonary interstitium and alveoli leads to pulmonary fibrosis, that is, the hypothesis of abnormal damage-repair of alveoli [2]. Up to now, the commonly used drugs in conventional treatment include anti-inflammatory, antifibrosis, cytokines, antioxidants, lung transplantation, and oxygen therapy [3]. The 2015 Guidelines [3] for the Diagnosis and Treatment of Idiopathic Interstitial Pneumonia pointed out that there was no evidence to prove which medicine can effectively treat IPF except lung transplantation [4]. The guidelines recommended pirfenidone and nintedanib which are not widely used in China due to the large side effects and the high price [3].

Therefore, it is an urgent problem to find new complementary and alternative medicine (CAM) to treat IPF. On the efficacy and value of traditional medicine as an alternative medicine, many scholars have conducted in-depth research in different fields such as immunology, biology, and chemistry [5]. In addition, many studies have been done on traditional medicine as a substitute for different disease groups [6, 7]. Thus, some scholars put forward that modern medicine should not be separated from traditional medicine; it is important to have traditional medicines as a complimentary medicine and to have them applied through “integrative approach,” that is, employing a personalized strategy by considering the patient's specific conditions; integrative medicine endeavors to apply all appropriate interventions from a whole set of science branches to bring back health [8]. In addition, some scholars believe that traditional medicine as CAM will become the mainstream of modern medical development [9].

IPF mostly belongs to the category of “pulmonary flaccidity,” “pulmonary arthralgia,” and “asthma syndrome” in traditional Chinese medicine [10]. The essence of it is considered as “deficiency of vital energy and blood stasis.” Deficiency in lung and kidney is the root, phlegm and blood stasis are the symptoms, and the blood stasis runs through the whole pathological process, therefore promoting that blood circulation and removing blood stasis is indispensable [10].

*Ligusticum wallichii*, or Chinese name Chuan xiong, has the functions of promoting blood circulation, removing blood stasis and stopping bleeding, eliminating the headache [11] and uncomfortable eyes caused by cold wind, regulating menstruation by descending into bloodstream, getting through the skin, and bypassing the limbs to facilitating the circulations. It is the medicine to relieve the stagnation of Qi and to smooth the blood. It has the effect of promoting blood circulation and the effect of removing blood stasis [12]. Ligustrazine is the main chemical component extracted from *Ligusticum wallichii* which is widely used in cardiovascular [13] and cerebrovascular diseases [14, 15], tumors [16], blood [17], gynecology [18], peripheral blood vessels [19], and other diseases. In recent years, its application in the field of fibrosis has also attracted considerable attention [20–22].

This study collected RCT of Ligustrazine in the treatment of IPF. The effectiveness and safety of Ligustrazine in the treatment of IPF was reviewed objectively by the systematic review method to provide evidence-based medical basis for its clinical application.

## 2. Methods

This study has been registered in PROSPERO (https://www.crd.york.ac.uk/prospero/), registration number CRD42020193626. The procedure of this protocol is based on PRISMA-P guidance [23].

### 2.1. Inclusion and Exclusion Criteria

#### 2.1.1. Types of Studies

Randomized controlled trial of Ligustrazine in the treatment of IPF in Chinese or English, whether or not blind, and allocation concealment were used.

#### 2.1.2. Types of Participants

It conforms to the authoritative standards established by the Society of Respiratory Diseases of the Chinese Medical Association in 2002 [24] or the American Thoracic Society/European Respiratory Society/Japanese Respiratory Society/Latin American Thoracic Society [1] in China and abroad. Its gender, age, race, and nationality are not limited, excluding serious diseases associated with other systems.

#### 2.1.3. Types of Intervention

In routine basic treatment, the experimental group is as follows: Ligustrazine treatment or Ligustrazine treatment given on the basis of the control group is not limited with dosage, dosage form, route, and method of administration. The control group is as follows: drugs (hormones, N-acetylcysteine, cyclophosphamide, azathioprine, etc.) and combined with the same routine treatment. The dosage and course of treatment are not limited. Routine treatment refers to oxygen therapy, lung function training, antibiotic therapy, and other measures.

#### 2.1.4. Types of Outcome Measures

Primary outcomes are as follows: total clinical effective rate: according to the clinical physiological X-ray (CRP) comprehensive scoring method developed by Watters et al. [25]: 1) dyspnea score 0–20 points, 2) respiratory frequency score 0–3 points, 3) lung rate score 0–6 points, 4) chest X-ray score 0–26 points, 5) FVC% score 0–12 points, 6) FEV_1_% score 0–3 points, 7) VC% score 0–10 points, and 8) arterial oxygen partial pressure (PaO_2_) score 0–20 points. The score of <−20 is significant, the score of <−20∼−10 is effective, the score of <−10∼ + 10 is stable, and the score of >10 is invalid. Total effective rate is as follows: [(number of obvious effective cases + number of effective cases)/total cases] × 100%.

Secondary outcomes are 1) pulmonary function, 2) arterial blood gas analysis, and 3) adverse reactions.

#### 2.1.5. Exclusion Criteria

Nonrandomized controlled trials, reviews, case reports, experimental studies, expert experience, incomplete information, and repeated publication were excluded. For multiple publications of the same study, only one with the largest sample size and the most complete information was retained.

### 2.2. Retrieval Strategy

PubMed, Embase, China National Knowledge Infrastructure Database (CNKI) (URL: WanFang” title = “https://www.cnki.net/),WanFang”>https://www.cnki.net/), WanFang Database (URL: http://www.wanfangdata.com.cn/index.html), and VIP Chinese Science and Technology Periodical Database (VIP) (URL: http://qikan.cqvip.com/) were retrieved by computer to find out a randomized controlled trial of Ligustrazine and its combination with routine therapy and control drugs and the same routine treatment for IPF. Theme words and keywords were retrieved combining with literature retrospective and manual retrieval methods. The search terms were as follows: “Idiopathic pulmonary fibrosis” or “Pulmonary fibrosisor” or “Pulmonary interstitial fibrosis” or “Interstitial lung disease” or “IPF”AND “Ligustrazine” or “Tetramethylpyrazine” or “TMP” or “Chuanxiongqin” or“Chuanxiong rhizome” or “Chuanxiong” or “Ligusticum wallichii” or “Ligustilide” AND “Randomized controlled trial” or “RCT”. The date of retrieval was self-built until August 2019. At the same time, Baidu Academy and Google Scholar search engines were used to supplement and retrieve relevant documents on the Internet.

### 2.3. Literature Screening and Data Extraction

Two evaluators (Xiaozheng Wu and Wen Li) independently screened the literature according to inclusion and exclusion criteria. After excluding studies that obviously did not meet inclusion criteria, they read the full text of studies that might meet inclusion criteria to confirm whether they really did and then cross-checked them. They discuss and resolve differences or handle them over to a third party (Yunzhi Chen) for decision when there was a disagreement. If the report was unknown or lacks information, they try to contact the author of the original text by sending emails to obtain further relevant data. The design of data extraction table generally follows the principle of “PICOST” (participants, interventions, comparisons, outcomes, study design, and time).

Data extraction contents include the following: general information, that is, research ID, author, title, publication status, report sources, and fund support; methodology information, that is, design, number of arms, random sequence generation, allocation concealment, blinding, incomplete outcome data, selective reporting, sample size calculation, and baseline comparability; participant information, that is, diagnostic criteria, inclusion criteria, exclusion criteria, setting, population, sample size, age, gender, and course of disease; intervention information, that is, name of intervention and comparation, types of TCM, dosage form, comparison, duration of treatment, and patient follow-up; outcomes; and adverse events.

### 2.4. Quality Evaluation of Primitive Research

The improved Jadad scale [26] was used as a quality evaluation criterion to evaluate the included literature.

### 2.5. Bias Risk Assessment of Primitive Research

The bias risk assessment tools recommended by Cochrane 5.1.0 assisted network were used to evaluate the study; contents evaluated included the following: 1) the generation of random allocation schemes, 2) hidden grouping, 3) the implementation of blind methods for patients and doctors, 4) the implementation of blind methods for results evaluation, 5) incomplete results data, 6) selective results report, and 7) other bias. The quality of the study results was evaluated one by one.

### 2.6. Statistical Analysis

The continuous data included in this study used the mean difference (MD), weighted mean differences (WMD), and 95% CI for curative effect analysis of statistics. The dichotomous outcomes included used the odds ratio (OR) value and 95% CI statistic for curative effect analysis. Subgroup analysis was carried out according to the possible heterogeneous factors. These factors were different measurement indicators, different medication time, and so on. When there was enough similarity between the data included in the subgroup (*P* > 0.1, *I*^2^ <50%), the fixed-effect model was used for the combined analysis. If there was clinical homogeneity but statistical heterogeneity in the subgroup, the randomized effect model was used to conduct a combined analysis. When heterogeneity originates from low-quality research, sensitivity analysis was carried out. Qualitative descriptions and analysis would be made for data that cannot be merged; publication bias was analyzed by funnel plots. All the statistical analysis above used Revman (Version 5.3. Copenhagen: The Nordic Cochrane Centre, The Cochrane Collaboration, 2014.) and Stata (Version 12.0, StataCorp, College Station, Texas) analysis software.

## 3. Results

### 3.1. Literature Retrieval Results

**Figure 1:**
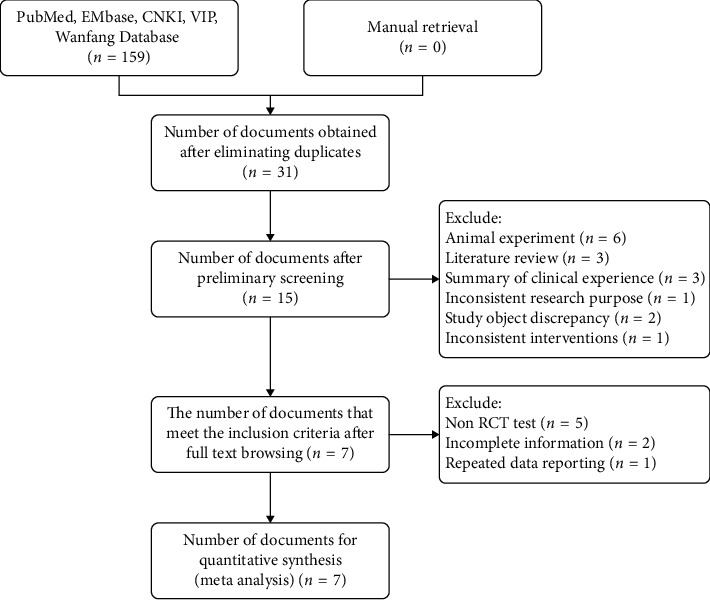
PRISMA literature screening flow chart.

One hundred and fifty-nine related literatures were initially detected from five databases, and seven studies [27–33] were finally included after screening step by step. All research is conducted in China. There were 366 patients in total, among which 196 were in the experimental group and 170 in the control group. According to the PRISMA statement [23], a literature screening flow chart is developed (see Figure 1, and the basic features of the study included are listed in Table 1).

### 3.2. Quality Assessment

The included seven studies were conducted in China, all of which were randomized controlled trials. They mentioned the use of randomized grouping method and did not describe the specific allocation hiding method or blind method. However, they all described the comparability of baseline data between the two groups and there were no incomplete data reports or data missing. The treatment methods and outcome indicators of the two groups were described in detail. The modified Jadad scale [26] was used to evaluate the 7 original studies, including 2 papers with 2 points [29, 32] and 5 papers with 1 point [27, 28, 30, 31, 33]. There were 7 low-quality studies. The results of methodological quality assessment are shown in Table 2.

### 3.3. The Cochrane Risk Bias Assessment Tool Was Used to Evaluate the Original Studies

**Figure 2:**
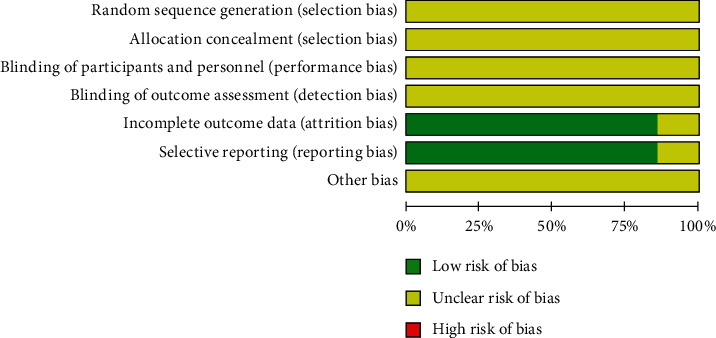
Bias risk percentage.

**Figure 3:**
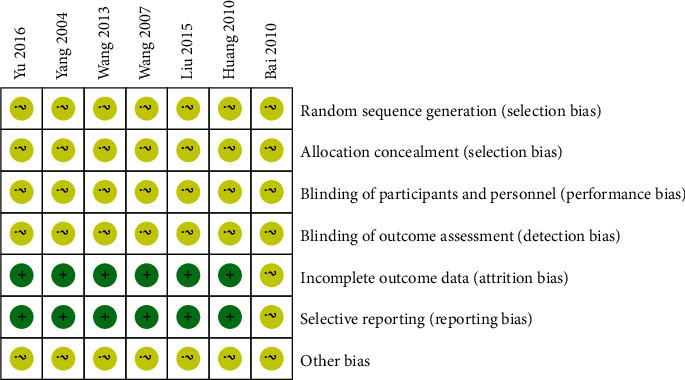
Bias risk summary chart.

The results showed that the proportion of low risk and moderate risk in random sequence generation included in the selection bias of the original studies was about 24% and 76%, respectively (see Figure 2). By analogy, there are certain selection, implementation, and measurement bias in the included study, and the literature bias statistics are shown in Figure 3.

### 3.4. Meta-Analysis

#### 3.4.1. Clinical Efficiency

**Figure 4:**
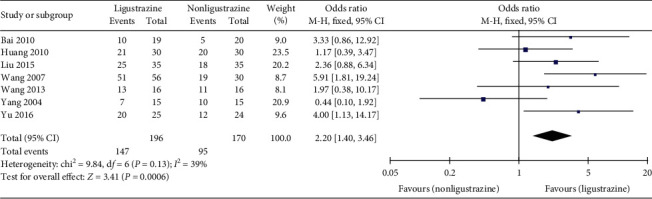
Clinical efficiency forest plot.

Seven trials [27–33] reported clinical efficacy in the studies. The effective cases in the combined treatment group and the control group were 196 and 170, respectively. There was no significant statistical heterogeneity among the studies (*P*=0.13, *I*^2^ = 39%). Therefore, fixed-effect model was used for combined analysis. The results showed that the clinical efficiency of Ligustrazine group was higher than that of control group ([OR] = 2.20, 95% CI [1.40, 3.46], *P*=0.0006); see Figure 4.

#### 3.4.2. FEV1/FVC %

**Figure 5:**
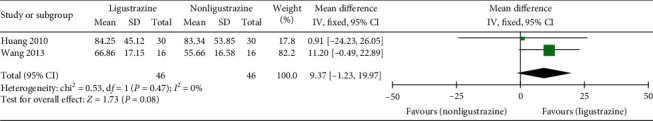
FEV_1_/FVC % forest plot.

Two trials [28, 31] reported the improvement of FEV_1_/FVC % in the included studies. The effective cases in the combined treatment group and the control group were 46 and 46, respectively. There was no statistical heterogeneity between the studies (*P*=0.47, *I*^2^ = 0%). Therefore, the fixed-effect model was used for combined analysis. The results showed that there was no significant difference in the improvement of FEV_1_/FVC % between Ligustrazine group and control group ([MD] = 9.37, 95% CI [−1.23, 19.97], *P*=0.08), as shown in Figure 5.

#### 3.4.3. VC %

**Figure 6:**
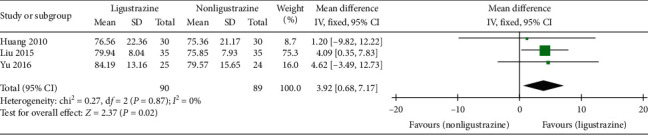
VC % forest plot.

In the included studies, three trials [28, 29, 33] reported the improvement of VC %. The effective cases in the combined treatment groups and the control groups were 90 cases and 89 cases, respectively. There was no statistical heterogeneity between the studies (*P*=0.87, *I*^2^ = 0%). Therefore, the fixed-effect model was used for combined analysis. The results showed that the improvement of VC % in Ligustrazine group was higher than that in control group ([MD] = 3.92, 95% CI [0.68, 7.17], *P*=0.02); see Figure 6.

#### 3.4.4. TLC %

**Figure 7:**
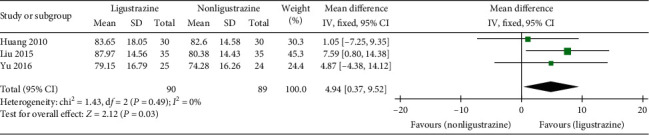
TLC % forest plot.

Three trials [28, 29, 33] reported the improvement of TLC % in the studies. The effective cases in the combined treatment groups and the control groups were 90 and 89, respectively. There was no statistical heterogeneity between the studies (*P*=0.49, *I*^2^ = 0%); therefore, fixed-effect model was used to analyze the results. The results showed that the improvement of TLC % in Ligustrazine group was higher than that in control group ([MD] = 4.94, 95% CI [0.37, 9.52], *P*=0.03); see Figure 7.

#### 3.4.5. DLCO %

**Figure 8:**
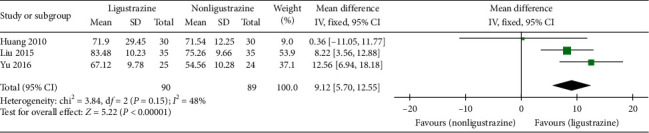
DLCO % forest plot.

Three trials [28, 29, 33] reported the improvement of DLCO % in the studies. The effective cases in the combined treatment groups and the control groups were 90 and 89, respectively. There was no substantial statistical heterogeneity between the studies (*P*=0.15, *I*^2^ = 48%). The results showed that the improvement of DLCO % in Ligustrazine group was higher than that in control group ([MD] = 9.12, 95% CI [5.70, 12.55], *P* < 0.00001), as shown in Figure 8.

#### 3.4.6. PaO_2_

**Figure 9:**
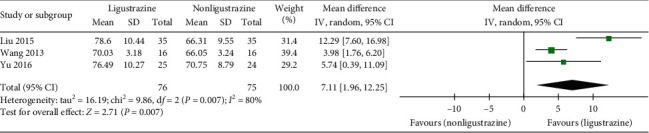
PaO_2_ forest plot.

In the included studies, three trials [29, 31, 33] reported the improvement of PaO_2_. The effective cases of the combined treatment groups and the control groups were 76 and 75, respectively. There was statistical heterogeneity among the studies (*P*=0.007, *I*^2^ = 80%). By analyzing the sources of heterogeneity, a conclusion could be drawn that the heterogeneity of the above four studies did not mainly come from clinical heterogeneity and methodological heterogeneity. Therefore, a random effect model was used to conduct a combined analysis. The results showed that the improvement of PaO_2_ in Ligustrazine group was higher than that in control group ([MD] = 7.11, 95% CI [1.96, 12.25], *P*=0.007); see Figure 9.

#### 3.4.7. PaCO_2_

**Figure 10:**
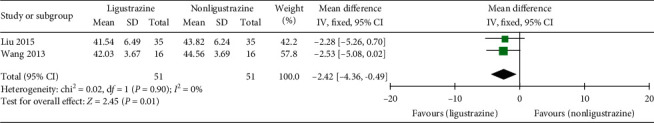
PaCO_2_ forest plot.

In the included studies, two trials [29, 31] reported the improvement of PaCO_2_. The effective cases in the combined treatment groups and the control groups were 51 and 51, respectively. There was no statistical heterogeneity among the studies (*P*=0.90, *I*^2^ = 0%); therefore, fixed-effect model was used for combined analysis. The results showed that the improvement of PaCO_2_ in Ligustrazine group was higher than that in control group ([MD] = −2.42, 95% CI [−4.36, −0.49], *P*=0.01); see Figure 10.

#### 3.4.8. Adverse Effects

In the included studies, 2 cases [29, 32] reported adverse effects, and 5 cases [27, 28, 30, 31, 33] showed no adverse effects. In one study [29], the control group had edema in 3 cases, hypertension in 1 case, hyperglycemia in 1 case, oral infection in 1 case, and bone pain in 1 case. In the treatment group, there were edema in 2 cases, hypertension in 2 cases, gastrointestinal bleeding in 2 cases, and depression in 1 case. Both groups were relieved after symptomatic treatment without affecting the follow-up treatment. There were no osteoporotic fracture, electrolyte disturbance, systemic serious infection, or obvious damage to liver and kidney function in both groups. In another study [32], there were no adverse effects in Ligustrazine group. Five cases in hormone group had gastric discomfort; however, they can adhere to the treatment after taking corresponding treatment.

**Figure 11:**
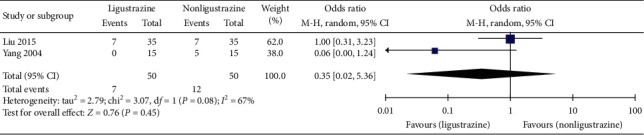
Adverse reactions forest plot.

Meta-analysis showed that there was no significant difference in adverse effects between Ligustrazine group and control group ([MD] = 0.35, 95% CI [0.02, 5.36], *P*=0.45), as shown in Figure 11.

#### 3.4.9. Influence Analysis

Influence analysis of the results of the clinical efficacy study was done and it showed that the minimum of all the study results was not lower than 1, indicating that removing any study will not make significant difference in the results. It is proved that the sensitivity of clinical effective quantity is low, and it has good stability and reliability, and the analysis result is stable and credible. See Table 3 and Figure 12 in Supplementary Materials.

Three methods were used to analyze PaO_2_. 1) The statistical analysis model of combined effect was changed, and the results showed that there was no statistical change in PaO_2_ analysis (*P*=0.02). 2) The research methods that are with large deviation in the data results were removed, and it showed that the effect of PaO_2_ analysis (*P* < 0.17, *I*^2^ = 40% *P*=0.02, *I*^2^ = 70%) had no significant change before and after the treatment and still had statistical significance (*P* < 0.0001). 3) The study was excluded one by one, the effect and *P* changes were observed, and the results show that, excluding any research literature, the *P* value of PaO_2_ is less than 0.05, but the effect does not change significantly. The three methods showed that PaO_2_ had low sensitivity and good stability and reliability, and the results were robust and credible.

#### 3.4.10. Publication Bias Analysis

**Figure 12:**
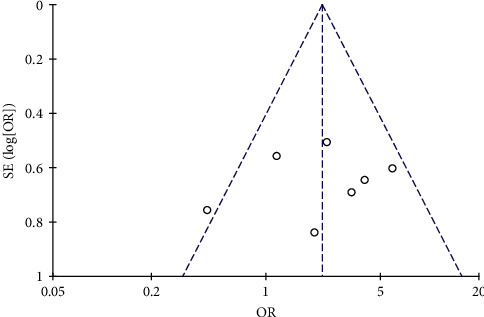
Inverted funnel chart of clinical efficacy.

Inverted funnel plot analysis was carried out in 7 cases of clinically effective studies. The results show that the inverted funnel is symmetrical, suggesting that there is no publication bias, and the results of this study are reliable; see Figure 12. Begg method and Egger method were used to detect the bias in the study. The results of Begg method showed that pr > *z* = 0.548, indicating that there was no significant bias in this study. The results of bias in Egger method showed that *P* > *t* = 0.668, indicating that there was no significant bias in this study. See Tables 4 and 5 and Figures 14 and 15 in Supplementary Materials.

## 4. Discussion

The purpose of this study is to systematically review the efficacy and safety of Ligustrazine in the treatment of IPF. The results showed that, compared with the control group, Ligustrazine could improve the clinical symptoms ([OR] =2.20, 95% CI [1.40, 3.46], *P*=0.0006), lung function (VC % [MD] = 3.92, 95% CI [0.68, 7.17], *P*=0.02) (TLC % [MD] =4.94, 95% CI [0.37, 9.52], *P*=0.03), the pulmonary diffusion function (DLCO % [MD] = 9.12, 95% CI [5.70, 12.55], *P* < 0.00001), and arterial blood gas analysis (PaO_2_ [MD] =7.11, 95% CI [1.96, 12.25*P*=0.007]) (PaCO_2_ [MD] = −2.42, 95% CI [−4.36, −0.49], *P*=0.01) of IPF patients, respectively. However, FEV_1_/FVC % ([MD] = 9.37, 95% CI [−1.23, 19.97], *P*=0.08) and adverse reactions ([MD] = 0.35, 95% CI [0.02, 5.36], *P*=0.45) were not significantly improved. It is suggested that Ligustrazine has some advantages and efficacy in the treatment of IPF as a complementary and alternative medicine. In addition, many experimental studies also revealed that Ligustrazine or prescriptions containing Ligustrazine has a good curative effect on IPF [34]. It can improve clinical symptom such as difficulty in breathing in patients with IPF, improve patients' life quality, pulmonary function, and pulmonary diffusion function [35, 36], inhibit collagen deposition of rat lung tissue, reduce the matrix metalloproteinase-1 (MMP-1) expression of matrix metalloproteinase-2 (MMP-2), restrain collagen-I (Col I) and collagen-III (Col-III), and reduce the accumulation of extracellular matrix [37–40]. Therefore, these data further prove the credibility of the results of this study and also indicate that Ligustrazine can act on IPF through multiple channels and targets.

### 4.1. Effectiveness and Safety Analysis

Meta-analysis showed that Ligustrazine was more effective to medicine in improving clinical efficiency, improving pulmonary function (TLC %, VC %, DLCO %), and blood gas analysis (PaO_2_, PaCO_2_), suggesting that Ligustrazine was effective and feasible in treating IPF. However, there are fewer data on the key indicators of improving lung function, such as FEV_1_/FVC %. The reason is estimated to be related to the insufficient sample size and the poor quality of research literature. It is necessary to further increase the number of high-quality RCT samples for demonstration. In addition, because there is no relevant mortality endpoint indicators reported in the study and no follow-up after treatment, it is impossible to judge whether there are differences between the experimental group and the control group in reducing mortality and other endpoint events.

Two cases of adverse reactions were reported in the study, but the symptoms were mild, the time was short, and the incidence was low. There were mainly some side effects of combination of the drugs. However, 5 cases of adverse reactions have not been mentioned in the research reports, and the safety of Ligustrazine in the treatment of IPF and the incidence of adverse reactions have not yet been proved. In addition, most studies have not been observed for very long time (<12 weeks), so the long-term safety cannot be evaluated. Longer-term high-quality RCTs are needed in the future.

### 4.2. Limitations of Inclusion

According to the inclusion and exclusion criteria in the study, all the 7 research papers included in this study described the outcome indicators in detail of the experimental group and control group, but there are still some problems. 1) Seven studies reported random methods, but they all only mentioned the methods and did not give a clear random method. None of the studies reported the use of allocation hiding and blind methods. All the studies did not report the treatment of missing interviews, which suggests a real possibility of the bias of the literature included in this study and a low evidence intensity. 2) Most of the studies only accounted the total clinical efficacy, the evaluation index was single, and the evaluation criteria of the results were different. Thus, there is a certain degree of clinical heterogeneity. 3) The sample size included in the study is small, most of them are single-center studies, and there are some clinical heterogeneity, such as course of treatment, course of disease, and intervention measures. All these factors will affect the reliability of the results. 4) Most of the studies did not statistically analyze the important observation indicators such as FEV_1_, FVC, 6-minute walking test (6MWD), St. George's respiratory questionnaire (SGRQ), and HRCT, which makes the evaluation index of the clinical efficacy single and the results of clinical efficacy questionable. 5) The literatures included in this study are all in China, no other countries, so it will be likely to produce a great linguistic bias and this may affect the conclusion and extrapolation of meta-analysis.

## 5. Conclusion

This systematic review and meta-analysis show that Ligustrazine has a certain clinical effect on IPF, which can improve the clinical symptoms, pulmonary function, and arterial blood gas analysis of IPF patients. And this result is meaningful in two aspects. First, it proves that Ligustrazine is a new alternative and complementary medicine for IPF. Its effect is better than that of conventional treatment. And in clinical practice, it can be considered as a new alternative and complementary medicine to be promoted and recommended for use in medical units in various countries in the world. Second, the obvious clinical efficacy of Ligustrazine proves and further confirms that the pathogenesis of IPF is “blood stasis,” and “blood stasis” runs through the whole pathological process of IPF; thus, Ligustrazine can treat IPF from the root of it by promoting blood circulation and removing blood stasis. It solved the difficult problem of conventional drug treatment of IPF; therefore, more research strength can be put in the treatment of the pathological mechanism of IPF for further exploration. However, the key indicators of improving lung function (FEV_1_/FVC %) and safety and adverse reactions need to be further analyzed and determined. And large sample, multicenter and randomized double-blind controlled trials are needed in the future to prove the efficacy and safety and long-term follow-up endpoint mortality of Ligustrazine and to have more reliable conclusions to benefit guiding clinical practice given that the related clinical research is few at present.

## Figures and Tables

**Table 1:** Basic features of the included study.

Studies	Sample (*n*)		Gender (male/female)	Age (Year)	Average course of disease (week)	Outcomes	Course (week)	Adverse reactions	Interventions	
	E	C							Experimental group	Control group
Bai 2010 [27]	19	20	23/16	68 ∼ 76	E : > 3; C : > 3	Clinical efficiency	52	Not described	Ligustrazine injection, prednisone, routine treatment	Prednisone, routine treatment

Huang 2010 [28]	30	30	E : 15/15; C : 14/16	E : 50∼70; C : 52∼71	E : 5∼ 54; C : 5∼ 60	Clinical efficiency, TLC %, DLCO %, VC %	12	Not described	Ligustrazine injection	Prednisone

Liu 2015 [29]	35	35	E : 29/6	E : 55.8 ± 12.9	——	Clinical efficiency, TLC %, DLCO %, VC %, PaO2	12	E : 7/35 (edema in 2 cases, hypertension in 2 cases, gastrointestinal bleeding in 2 cases and depression in 1 case)	Ligustrazine injection, budesonide, routine treatment	Budesonide, routine treatment
			C : 28/7	C : 53.6 ± 11.7	——			C : 7/35 (edema in 3 cases, hypertension in 1 case, hyperglycemia in 1 case, oral infection in 1 case, and bone pain in 1 case)		

Wang 2007 [30]	56	30	E : 36/20	26–58	2–12	Clinical efficiency	Unclear	Not described	Ligustrazine injection	Prednisone
			C : 18/12							
Wang 2013 [31]	16	16	16/16	42 ∼ 70	36–96	Clinical efficiency, PaO2	2	Not described	Ligustrazine injection, prednisone, routine treatment	Prednisone, routine treatment

Yang 2004 [32]	15	15	E : 11/4	E : 53.15 ± 12.2	——	Clinical efficiency	8	E : 0/15	Ligustrazine injection, routine treatment	Prednisone, routine treatment
			C : 10/5	C 55. 09 ± 12.4	——			C : 5/15 (gastric discomfort)		
Yu 2016 [33]	25	24	27/22	55–82	3–18	Clinical efficiency, TLC %, DLCO %, VC %, PaO2	4	Not described	Ligustrazine injection, methylprednisolone, routine treatment	Methylprednisolone, routine treatment

Note: E: experimental group; C: control group.

**Table 2:** Quality evaluation of original research.

Studies	Randomization method	Allocation concealment	Blind method	Loss to follow-up	Baseline comparability	Jadad score
Bai 2010 [27]	Random	Not described	Not described	Not described	No significant difference	1
Huang 2010 [28]	Random	Not described	Not described	Not described	No significant difference	1
Liu 2015 [29]	Random	Not described	Not described	Adverse reactions	No significant difference	2
Wang 2007 [30]	Random	Not described	Not described	Not described	No significant difference	1
Wang 2013 [31]	Random	Not described	Not described	Not described	No significant difference	1
Yang 2004 [32]	Random	Not described	Not described	Adverse reactions	No significant difference	2
Yu 2016 [33]	Random	Not described	Not described	Not described	No significant difference	1

## Data Availability

This paper is a review and the data are from published literature; it is valid.

## Supplementary Materials

Supplementary Materials
*Supplementary figures.* Figure 12: influence analysis results of clinical efficacy. Figure 14: the funnel chart generated by Egger method. Figure 15: the funnel chart of bias generation detected by Begg rank correlation. Supplementary tables: Table 3: influence analysis results data of clinical efficacy. Table 4: detection results of bias in the study by Begg method.

## References

[1] Raghu G., Collard H. R., Egan J. J. (2011). An official ATS/ERS/JRS/ALAT statement: idiopathic pulmonary fibrosis: evidence-based guidelines for diagnosis and management. *American Journal of Respiratory and Critical Care Medicine*.

[2] Wolter P. J., Collard H. R., Jones K. D. (2014). Pathogenesis of idiopathic pulmonary fibrosis. *Annual Review of Pathology: Mechanisms of Disease*.

[3] Raghu G., Rochwerg B., Zhang Y. (2015). An official ATS/ERS/JRS/ALAT clinical practice guideline: treatment of idiopathic pulmonary fibrosis. An update of the 2011 clinical practice guideline. *American Journal of Respiratory and Critical Care Medicine*.

[4] Olsen A. L., Swigris J. J., Lezotte D. C., Norris J. M., Wilson C. G. (2007). Mortality from pulmonary fibrosis increased in the United States from 1992 to 2003. *American Journal of Respiratory and Critical Care Medicine*.

[5] Cooper E. L. (2005). CAM, eCAM, bioprospecting: the 21st century pyramid. *Evidence-Based Complementary and Alternative Medicine*.

[6] Khiveh A., Hashempur M. H., Shakiba M. (2017). Effects of rhubarb (Rheum ribes L.) syrup on dysenteric diarrhea in children: a randomized, double-blind, placebo-controlled trial. *Journal of Integrative Medicine*.

[7] Afrasiabian F., Mirabzadeh Ardakani M., Rahmani K. (2019). Aloysia citriodora palau (lemon verbena) for insomnia patients: a randomized, double-blind, placebo-controlled clinical trial of efficacy and safety. *Phytotherapy Research*.

[8] Daneshfard B., Sanaye M. R., Nimrouzi M. (2019). Prolegomena to a true integrative medical paradigm. *Alternative Therapies in Health and Medicine*.

[9] Shmueli A., Shuval J. (2007). Are users of complementary and alternative medicine sicker than non-users?. *Evidence-Based Complementary and Alternative Medicine*.

[10] Cui H.-S., Wang Q. (2004). Four elements of differentiation and treatment of pulmonary interstitial fibrosis. *New Chinese Medicine*.

[11] Wang G.-X., Zhao J.-H., Wang Y.-B. (2020). Clinical effect of ibuprofen combined with chuanxiong tea decoction in the treatment of migraine. *Journal of Clinical Rational Drug Use*.

[12] Zhang W.-H. (2015). Pharmacology and clinical application of ligusticum chuanxiong. *Chinese Health Standard Management*.

[13] Qian C. (2014). Pharmacological action of ligustrazine on cardiovascular system. *Inner Mongolia Chinese Medicine*.

[14] Zhao C. (2020). Clinical efficacy evaluation of taoren chuanxiong decoction in treatment of sequela of cerebral hemorrhage in 45 cases. *Chinese Practical Medicine*.

[15] Liu Y. (2020). Observation of efficacy of ligustrazine combined with conventional western medicine in the treatment of ischemic cerebrovascular disease. *Chinese Journal of Integrated Traditional and Western Medicine*.

[16] Wang D.-Y., Deng B., Deng Y.-Y. (2020). Effects of ligustrazine combined with cisplatin on expression of stromal cell derived factor-1, apoptosis inhibitor protein factor and matrix metalloproteinase in elderly patients with lung cancer. *Anhui Medicine*.

[17] Sun Y.-L. (202). Effect of Buyang Huanwu decoction combined with ligustrazine on hemorheology indexes and nerve function in patients with acute cerebral infarction. *Medical Equipment*.

[18] Feng R., Guo L., Pu Z.-H. (2020). Chemical composition of ligusticum chuanxiong and its inhibitory effect on uterine smooth muscle contraction. *Chinese Patent Medicine*.

[19] Li F.-F., Zhang Q. (2020). Research progress of protective mechanism of ligustrazine on vascular endothelial injury. *Chinese Medical Journal*.

[20] Hu Y.-H., Wang X., Fang J.-Y. (2020). Protective effect of Ginseng - panax notoginseng-ligusticum chuanxiong extract on myocardial fibrosis in diabetic mice. *Chinese Journal of Experimental Formulology*.

[21] He P.-F., Gao M., Zhang Y. (2019). Chinese herbal compound against renal interstitial fibrosis. *Yunnan Journal of Traditional Chinese Medicine*.

[22] Tang P., Li Y. (2019). Study on the relationship between single Chinese medicine and autophagy on hepatic fibrosis. *Journal of Public Science and Technology*.

[23] Moher D., Shamseer L., Clarke M. (2015). Preferred reporting items for systematic review and meta-analysis protocols (PRISMA-P) 2015 statement. *Systematic Reviews*.

[24] Society of Respiratory Diseases, Chinese Medical Association (2002). Guidelines for the diagnosis and treatment of idiopathic pulmonary (interstitial) fibrosis (draft). *Chinese Journal of Tuberculosis and Respiration*.

[25] Watters L. C., King T. E., Schwarz M. I., Waldron J. A., Stanford R. E., Cherniack R. M. (1986). A clinical, radiographic, and physiologic scoring system for the longitudinal assessment of patients with idiopathic pulmonary fibrosis 1–3. *American Review of Respiratory Disease*.

[26] Jadad A. R., Moore R. A., Carroll D. (1996). Assessing the quality of reports of randomized clinical trials: is blinding necessary?. *Controlled Clinical Trials*.

[27] Bai S.-P. (2010). Clinical observation of 36 cases of chronic idiopathic pulmonary fibrosis treated by ligusticum chuanxiong. *China Medical Frontier*.

[28] Huang Y.-L. (2010). Clinical observation of 30 cases of idiopathic pulmonary fibrosis treated by huang yanling and ligustrazine injection. *Hebei Traditional Chinese Medicine*.

[29] Liu Y. (2015). Observation on the efficacy of ligustrazine combined with nebulized budesonide inhalation in the treatment of idiopathic pulmonary fibrosis. *Journal of Modern Integrated Chinese and Western Medicine*.

[30] Wang W.-P. (2007). Observation on the therapeutic effect of Ligustrazine on idiopathic pulmonary interstitial fibrosis. *Shandong Journal of Traditional Chinese Medicine*.

[31] Wang T. (2013). Observation on the efficacy of Ligustrazine Injection in the treatment of patients with idiopathic pulmonary fibrosis. *Inner Mongolia Traditional Chinese Medicine*.

[32] Yang X.-Z. (2004). Ambroxol hydrochloride combined with ligustrazine in the treatment of idiopathic pulmonary fibrosis. *Journal of Practical Diagnosis and Treatment*.

[33] Yu Z. (2016). 25 cases of idiopathic pulmonary interstitial fibrosis treated with Ligustrazine. *Journal of Heze Medical College*.

[34] Liu W.-f., Shi L.-Q. (2012). Clinical study of yiqi yangyin prescription in the treatment of idiopathic pulmonary fibrosis with deficiency of qi and yin. *Journal of Beijing University of Traditional Chinese Medicine: Clinical Edition of Traditional Chinese Medicine*.

[35] Xin D.-Y., Fu J.-J. (2015). Clinical observation on treatment of idiopathic pulmonary interstitial fibrosis of qi deficiency and blood stasis type with dimensional xiaozheng decoction. *World Traditional Chinese Medicine*.

[36] Li Y., Wang X.-J., Zhang S.-S. (2013). Observation of curative effect of Feiwei Granule on 61 cases of pulmonary interstitial fibrosis. *Journal of Traditional Chinese Medicine*.

[37] Jiang Y., Li W., Chen M. (2008). Effect of Ligustrazine on CTGF expression and collagen deposition in rats with pulmonary fibrosis. *Chinese Journal of General Medicine*.

[38] Cheng W., Duan F., Li S.-C. (2011). MMP expression of ligustrazine in the prevention and treatment of idiopathic pulmonary fibrosis. *Medical Research and Education*.

[39] Wang Z.-X., Sun X.-F., Li S.-C. (2010). Effect of ligustrazine on the expression of MMP-2 in bleomycin induced pulmonary fibrosis rats. *Medical Research and Education*.

[40] Li J., Huang M., Wu F. (2007). Intervention of ligustrazine on pathomorphology and extracellular matrix of bleomycin-induced pulmonary fibrosis in rats. *Chinese Journal of Traditional Chinese Medicine*.

