# Supplementary material for: Meta-Analysis of Clinical Efficacy and Safety of Ligustrazine in the Treatment of Idiopathic Pulmonary Fibrosis
Source: Evid Based Complement Alternat Med. 2020 Nov 6;2020:2416132. doi: 10.1155/2020/2416132 (PMC7665923; doi:10.1155/2020/2416132)
Supplement: Supplementary Materials — Supplementary figures. Figure 12: influence analysis results of clinical efficacy. Figure 14: the funnel chart generated by Egger method. Figure 15: the funnel chart of bias generation detected by Begg rank correlation. Supplementary tables: Table 3: influence analysis results data of clinical efficacy. Table 4: detection results of bias in the study by Begg method. [file 2416132.f1.doc]

**Table 3 Influence analysis results data of clinical efficacy**

| Study omitted | Estimate | [95% Conf. Interval] | |
| --- | --- | --- | --- |
| Bai (2010) | 2.0856652 | 1.2900378 | 3.3719943 |
| Huang (2010) | 2.5159843 | 1.5243059 | 4.1528254 |
| Liu (2015) | 2.1574986 | 1.2972244 | 3.5882766 |
| Wang (2007) | 1.8470165 | 1.1267004 | 3.0278409 |
| Wang (2013) | 2.2186913 | 1.3858112 | 3.5521371 |
| Yang (2004) | 2.6641819 | 1.6408398 | 4.3257513 |
| Yu (2016) | 2.007206 | 1.2344569 | 3.2636831 |
| Combined | 2.1985558 | 1.3985404 | 3.4562088 |

**Figure 12 Influence analysis results of clinical efficacy**

**Table 4 Detection results of bias in the study by Begg method**

| Begg's Test | |
| --- | --- |
| adj. Kendall's Score (P-Q) | -5 |
| Std. Dev. of Score | 6.66 |
| Number of Studies | 7 |
| z | -0.75 |
| Pr > |z| | 0.453 |
| z | 0.60 (continuity corrected) |
| Pr > |z| | 0.548 (continuity corrected) |

**Table 5 Detection results of bias in the study by egger method**

| Egger's test | | | | | | |
| --- | --- | --- | --- | --- | --- | --- |
| Std_Eff | Coef. | Std. Err. | t | P>|t| | [95% Conf. Interval] | |
| slope | 1.725289 | 2.055989 | 0.84 | 0.440 | -3.559798 | 7.010376 |
| bias | -1.482549 | 3.260461 | -0.45 | 0.668 | -9.863832 | 6.898733 |

**Figure 14 The funnel chart generated by egger method**

**Figure 15 The funnel chart of bias generation detected by Begg rank correlation**
